# Rapid Environmental Change over the Past Decade Revealed by Isotopic Analysis of the California Mussel in the Northeast Pacific

**DOI:** 10.1371/journal.pone.0025766

**Published:** 2011-10-03

**Authors:** Catherine A. Pfister, Sophie J. McCoy, J. Timothy Wootton, Pamela A. Martin, Albert S. Colman, David Archer

**Affiliations:** 1 Department of Ecology and Evolution, University of Chicago, Chicago, Illinois, United States of America; 2 Department of Geophysical Sciences, University of Chicago, Chicago, Illinois, United States of America; National Institute of Water & Atmospheric Research, New Zealand

## Abstract

The anthropogenic input of fossil fuel carbon into the atmosphere results in increased carbon dioxide (CO_2_) into the oceans, a process that lowers seawater pH, decreases alkalinity and can inhibit the production of shell material. Corrosive water has recently been documented in the northeast Pacific, along with a rapid decline in seawater pH over the past decade. A lack of instrumentation prior to the 1990s means that we have no indication whether these carbon cycle changes have precedence or are a response to recent anthropogenic CO_2_ inputs. We analyzed stable carbon and oxygen isotopes (δ^13^C, δ^18^O) of decade-old California mussel shells *(Mytilus californianus*) in the context of an instrumental seawater record of the same length. We further compared modern shells to shells from 1000 to 1340 years BP and from the 1960s to the present and show declines in the δ^13^C of modern shells that have no historical precedent. Our finding of decline in another shelled mollusk (limpet) and our extensive environmental data show that these δ^13^C declines are unexplained by changes to the coastal food web, upwelling regime, or local circulation. Our observed decline in shell δ^13^C parallels other signs of rapid changes to the nearshore carbon cycle in the Pacific, including a decline in pH that is an order of magnitude greater than predicted by an equilibrium response to rising atmospheric CO_2_, the presence of low pH water throughout the region, and a record of a similarly steep decline in δ^13^C in algae in the Gulf of Alaska. These unprecedented changes and the lack of a clear causal variable underscores the need for better quantifying carbon dynamics in nearshore environments.

## Introduction

Carbon dioxide-induced changes to the ocean carbon cycle have the potential to dramatically change the biology of the ocean [1]. There are several reports of precipitous declines in coastal seawater pH over the past 15 years [2], [3], [4], [5], but it is impossible to know whether these declines are anomalous or part of a natural fluctuation. The biological response will depend on whether organisms have experienced such pH levels in recent decades or centuries, as well as the rate of future change.

The chemical composition and growth patterns of shells of marine organisms can be used to infer the physical and chemical features of the ocean on both recent and paleontological time scales [6], [7], [8], [9]. Shells can provide proxy chemical records spanning longer time intervals than the instrumental record. The relative proportions of light (^12^C) and heavy (^13^C) stable isotopes of carbon (δ^13^C) in calcium carbonate-based shells can indicate carbon sources. Respiration of organic matter in subsurface ocean waters imprints intermediate and deep waters with isotopically light respired dissolved inorganic carbon (DIC), resulting in an inverse relationship between the intensity of upwelling and δ^13^C in the shell of the California mussel (*Mytilus californianus)* in southern California [6]. Shell material should also become less enriched in ^13^C due to inputs of isotopically lighter carbon from fossil fuel sources (the Suess effect: [10], [11]), lowering the relative abundance of ^13^C in atmospheric CO_2_ and surface ocean DIC through time. Stable isotope ratios of oxygen reflect water temperatures, with higher δ^18^O values indicating colder water [6], [9], [12], such as water upwelled from lower depths. Thus, carbon and oxygen isotopes of calcareous material are often used to reconstruct environmental data in the ocean [8].

Tatoosh Island (48.32°N, 124.74°W), 0.7 km off the northwestern tip of Washington State, USA, has been the site of extensive ecological investigation for five decades. Seawater data collected by a Hydrolab DataSonde (Hach Company, Loveland, CO) since 2000 show that the ocean at this site has undergone a sustained decline in pH over the past decade [2] at a rate that is an order of magnitude greater than expected based on model predictions [13] and the equilibrium response to rising atmospheric CO_2_ concentration. Field studies in the offshore waters of Washington [3] and throughout Puget Sound [4] have also found pH values in the surface ocean that were not anticipated for decades. At Tatoosh Island, there is no definitive explanation for the speed of this decline; aside from atmospheric CO_2_, there was little evidence for systematic changes in potential explanatory variables, including upwelling, chl *a*, temperature, salinity, and the Pacific Decadal Oscillation (PDO) [2]. Here we show that the shells of a cohort of mussels that recruited ∼30 m from the instrument in the year prior to its deployment preserved a record of a changing carbon cycle. Additionally, archival *M. californianus* shells exist due to the cadre of researchers that have studied at Tatoosh Island over the past several decades and also from the Makah tribe who have utilized the island for millennia [14]. We thus present isotopic analyses of historical shell sources from the same site spanning the past 1300 years, which demonstrate that recent carbon isotopic compositions of shells are historically unprecedented.

## Results

**Figure 1:**
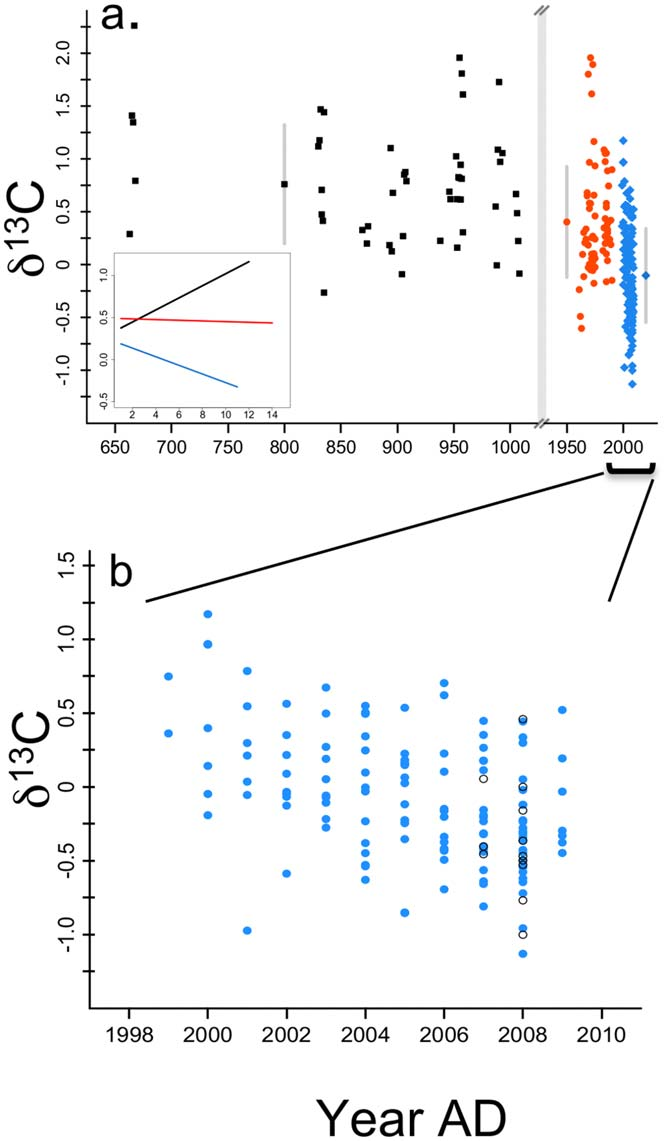
The δ^13^C for inner shell material of *M. californianus* from Tatoosh Island. a. The δ^13^C (‰, PDB scale) for inner shell material from 663 to 2009 AD. The data are divided into Native American midden shells (black, n = 11), archival shells (red) collected ∼1975 (n = 5), 1986 (n = 7), 1990 (n = 3), and modern shells (blue) collected in 2009 and 2010 (n = 56). Note that the archival shells from the 1970s do not have an exact collection year; we have assigned them 1975. Means for each of these 3 groups are shown with standard error bars. Inset figure shows the relationship between δ^13^C and age (in years) using a linear mixed effects model for each of the 3 shell groupings. There was no relationship between δ^13^C and mussel age for archival shells (coefficient = 0.003 ‰ yr^−1^, df = 49, p = 0.823) while midden mussels showed a positive relationship (0.064 ‰ yr^−1^, df = 38, p = 0.041). b. An expanded scale for the relationship between δ^13^C and year for modern shells only with small, young shells (38.8–52.6, n = 9) designated with a “o”. A linear mixed effects model estimated a significant negative slope of −0.071 ‰ yr^−1^ (se = 0.011, df = 85, p<0.001).

**Figure 2:**
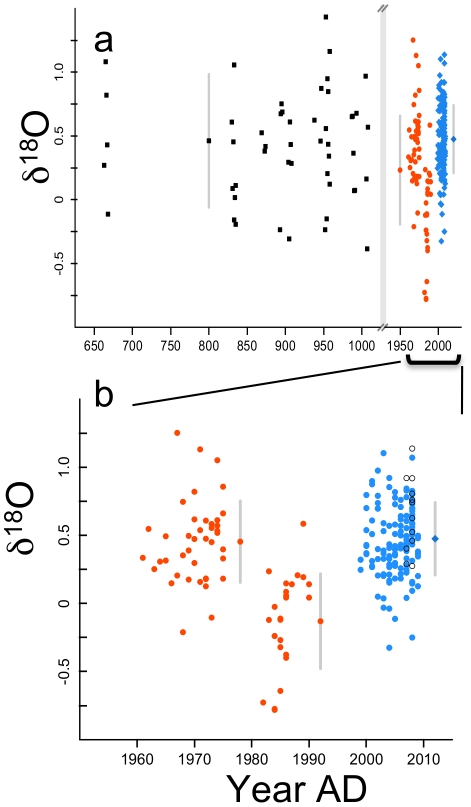
The δ^18^O for inner shell material of the same *M. californianus* from Tatoosh Island. a. The δ^18^O (‰, PDB scale) for inner shell material from 663 to 2009 AD with shells as shown in Fig 1. Figure key is identical to Fig 1. b. An expanded scale for archival and modern shells. The slope of δ^18^O with year for modern shells is not significant (0.006 ‰ yr^−1^, se = 0.008, df = 85, p = 0.469).

The modern *M. californianus* shells from Tatoosh Island were aged as far back as 1999 providing historical material for as much as 11 years per individual. The archival shell material spans from the 1960s through 1990, while midden shells were dated back to 663–1008 AD, dates consistent with previous estimates of human occupation [14]. The δ^13^C values (PDB scale) ranged from −1.13 to 1.17 ‰ from 663 A.D. until 2009. The mean value of δ^13^C has declined into the present with a decrease of 0.36 ‰ from the midden to the archival shells and an additional 0.53 ‰ decline for δ^13^C in modern shells (Figure 1a, ANOVA and Tukey HSD, F_2,289_ = 81.32, p<0.001). The δ^18^O of *M. californianus* showed a different pattern, with midden and modern shells having similar means, while the archival shells had a lower mean δ^18^O (Fig 2a, ANOVA and Tukey HSD, F_2,289_ = 11.77, p<0.001), perhaps recording warming during the 1983–1984 ENSO event. We further verified that the shells record oceanic events by using the Kim and O'Neil equation [15] modified by Ford [9] to predict seawater temperature from our modern shell material. Using our measured δ^18^O at Tatoosh in 2009 (−0.50‰, n = 17 from May to August) and 2010 (−0.87‰, n = 20), all modern shell δ^18^O values generated a range of temperatures from 5.9 to 14.0°C, with a mean of 10.4 and 8.8 using 2009 and 2010 δ^18^O measurements, and were thus were highly comparable to our mean measured seawater temperatures (Table 1).

**Table 1:** The isotopes of Carbon and Oxygen (‰, PDB scale) in the shells of *M. californianus* and environmental variables at Tatoosh and vicinity as a yearly average.

Year	δ^13^C	δ^18^O	SST§	pH§	DO§	Chl a§	NO_3_^−^	NH_4_	PO_4_^−^	SiO_2_	UI	PDO	SST_CE_	CO_2_
1999	0.56	0.28					20.04	1.63	1.97	37.08	−41.42	−1.063	12.19	368.1
2000	0.49	0.52	9.99	8.39	6.52	16.39	18.81	1.51	1.93	38.90	−25.00	−0.590	11.39	369.4
2001	0.12	0.54	9.70	8.27	6.99	17.09	18.27	2.46	1.97	40.36	−29.33	−0.563	11.60	371.1
2002	0.04	0.54	9.37	8.42	7.16	12.63	21.15	1.77	2.10	42.39	−29.17	0.221	11.65	373.2
2003	0.10	0.37	9.78	8.32			14.83	2.44	1.75	35.24	−41.50	0.969	11.87	375.8
2004	−0.04	0.33	9.84	7.77			18.69	4.19	2.14	36.86	−17.58	0.345	12.18	377.5
2005	−0.15	0.43	10.73	8.22			14.40	2.12	1.51	25.67	−11.58	0.375	11.86	379.8
2006	−0.12	0.48	9.77	7.97	9.10	38.92	17.89	1.47	1.55	36.03	−8.42	0.191	12.72	381.8
2007	−0.21	0.47	9.96	8.18			22.70	2.20	2.03	40.38	−7.08	−0.196	10.51	383.7
2008	−0.34	0.59	9.00	7.79	8.34	8.40	20.21	3.16	2.14	38.27	−1.92	−1.293	10.74	385.6
2009	−0.11	0.34	9.34	7.95	8.86	124.82	24.51	2.94	2.45	49.81	−11.50	−0.613	11.10	387.3

§ data from *in situ* Hydrolab instrument, nutrient data were measured at 10 sites on Tatoosh. UI, PDO, SST at Cape Elizabeth buoy, and atmospheric CO_2_ are means for the entire year.

### Patterns and Environmental Correlates in Modern Mussels

The δ^13^C of modern shells at Tatoosh declined significantly at a rate of −0.071 ‰ yr^−1^ over an 11-year period (Figure 1b, linear mixed effects model, se = .011, n = 53, df = 85, p<0.001). Although mussels were collected from 4 sites on Tatoosh in 2009, site was not a significant explanatory variable for δ^13^C measurements (Likelihood ratio Test, χ_1_^2^ = 2.8, p = 0.10) We also found no effect of shell age on δ^13^C when we compared the δ^13^C in 2007 and 2008 for large (129.3 – 158.0 mm length) versus small (38.8–52.6 mm, n = 9) mussels collected in 2009 (ANOVA, p = 0.547, Fig 1b). In contrast to δ^13^C, there was no systematic change in the δ^18^O from these same modern *M. californianus* shells, although the mean differed among years (Fig 2b, linear model, slope = 0.006, se = 0.008, df = 85, p = 0.469). As with δ^13^C, there was no effect of mussel size (small vs large) on the mean δ^18^O (ANOVA, p = 0.091, Fig 2b). The *M. californianus* collected by E. Sanford in August 2001 were 2–3 years old and thus extended back only to 1999, overlapping with our decade-old shells collected in 2009. Although they did not allow us to extend an archival record prior to 1999, they further demonstrated concordance with our 2009 shells; there were no differences in the δ^13^C values in 1999, 2000, and 2001 (p = 0.839) nor with δ^18^O values (p = 0.288) with these shells of different ages. The inclusion of these 2001 shells negligibly affected our estimates of δ^13^C with year, resulting in a slope of −0.069 vs −0.071 ‰ yr^−1^ without these 2001 shells.

Carbon and oxygen isotopes of mussels showed some strong relationships with environmental data. The δ^13^C of mussels exhibited a significant negative relationship with the annually averaged upwelling index (UI) and a significant positive relationship (and the lowest AIC scores) with seawater pH and atmospheric CO_2_ (Table 2) when these variables were tested independently. A linear mixed effect model using these three independently significant variables to explain the decline in δ^13^C was favored in model selection over any single variable model with a Likelihood Ratio Test (130.4 vs 139.8–147.6, Table 2) and thus strongly suggests that shells are responsive to carbon chemistry. All other environmental variables tested, including the UI (from April to September only), the PDO, silicate, nitrate and the seawater temperature at Tatoosh, were not associated with shell δ^13^C (Table 2).

**Table 2:** The results of linear mixed effects models testing explanatory variables for carbon and oxygen isotopes in *Mytilus californianus*.

Environmental Variable	δ^13^C		δ^18^O	
	Coefficient	AIC	Coefficient	AIC
Upwelling Index (year)	−0.015**	155.7	0.003	47.3
Upwelling Index (Apr-Sep)	−0.001	163.6	0.003	46.5
PDO (year)	0.007	159.2	−0.088*	38.5
SST	−0.003	159.4	−0.082*	**37.0**
Water temperature	0.129	154.1	−0.090	40.73
pH	0.586*	148.9	-	
nitrate	0.016	161.8	-	
silicate	0.000	163.5	-	
Atmospheric CO_2_	−0.035**	147.8	-	
salinity			−0.005	42.8
**All 3 significant variables**		**146.5**		

a. δ^13^C (‰, PDB scale) and b. δ^18^O (‰, PDB scale) in *M. californianus* from 1999(or 2000)–2009. The coefficient and AIC are based on a linear mixed effects model where the individual shell was the random effect. Environmental variables were first fit in isolation. The best fit model (bold) to the δ^13^C data included all 3 significant variables: UI for the year, pH and atmospheric CO_2_. Sample size ranged from 53 to 56 individual shells over 11 years. ** p<0.001, *p<0.05.

The PDO, calculated as either an annual average or averaged from April to September, was the only variable associated with the δ^18^O of mussels, showing a consistent negative relationship. The lower δ^18^O values in the archival shells may reflect the timing of the 1983/84 ENSO event, where surface temperatures warmed when upwelling weakened [16]. No significant variation in the δ^18^O of *M. californianus* shells was explained by salinity, seawater temperature, or the UI (Table 2). The lack of correlation with local mean annual temperature or salinity might arise because our instrumental data are not year-round, as is the PDO. Salinity also varies relatively little at Tatoosh on an interannual basis. The stronger correlation of both the UI and PDO with δ^13^C and δ^18^O when averaged over the entire year, rather than only April to September, suggest that there is continuous shell growth and that conditions over the entire year are recorded in the shell.

### Patterns in Archival and Midden Mussels

The δ^13^C values of archival and midden *M. californianus* were significantly higher than modern shells and showed no evidence of the declining trend in δ^13^C that characterized modern shells. Over the 14 individual mussels we analyzed spanning this period, the coefficient for an effect of year (0.006 ‰ yr^−1^) did not differ significantly from 0 (df = 49, p = 0.538) and was positive. The 11 *M. californianus* shells from Native American middens on Tatoosh also showed no significant year trend for δ^13^C (−0.001 ‰ yr^−1^, df = 38, p = 0.354). However, in the case of midden shells, the time span is several centuries. We thus also analyzed shells for a pattern and found a positive relationship (0.064 ‰ yr^−1^, df = 38, p = 0.041). Thus, the pattern of declining δ^13^C over the past decade at Tatoosh Island has not been observed at any other time over past decades and centuries.

The pattern at Tatoosh was also observed at another locale 85 km inside the Strait of Juan de Fuca at Observatory Point, WA (48.264°N, 124.248°W). Although the Observatory Point mussels were not as old as the modern Tatoosh mussels, with a record extending only to 2002, the shell δ^13^C values did not differ from those at Tatoosh from 2002−2008 (ANOVA, slope = −0.037 ‰ yr^−1^ (se = 0.034), site effect: p =  0.224, site by year interaction: p = 0.614). There was also no difference in the δ^18^O pattern through time between these two sites (ANOVA, site effect: p = 0.967).

## Discussion

The rate of decline in mussel shell δ^13^C from 1999−2009 was faster than can be explained by equilibration with the rising CO_2_ concentration of the atmosphere and the increasing contribution of isotopically light fossil fuel carbon (the Suess effect). The relationship between δ^13^C in atmospheric CO_2_ and the amount of CO_2_ in the atmosphere, δ^13^C  =  −0.0147[CO_2_]-2.60, predicts a δ^13^C rate of change of −0.0282 ‰ yr^−1^ [17], assuming rapid equilibration between the surface ocean and the atmosphere. Other calculations for seawater DIC estimate a global ocean average decline of −0.015 ‰ yr^−1^ in δ^13^C_DIC_ [18]. Both are much less than our observed decline in δ^13^C of −0.071 ‰ yr^−1^; the observed shell δ^13^C decline is consistent in sign with the observed 10-year decline in pH at Tatoosh Island [2], although the pH decrease was even greater, over 13 times stronger than can be explained by equilibrium with the rising atmospheric CO_2_ over this time period. The carbon chemistry of the surface waters is thus changing much more quickly than can be explained by simple immediate forcing from atmospheric CO_2_.

### Hypotheses for the Rapid δ^13^C Decline in Mussel Shells

We address three hypotheses for the shell δ^13^C decline that is 2.5 times greater than expected based on atmospheric CO_2_ levels and the assumption that atmospheric CO_2_ is in equilibrium with the pCO_2_ of the surface ocean (e.g. [19]). First, the food web has shifted from a carbon source that is heavier to one that is lighter, a possibility if phytoplankton has replaced kelp and benthic microalgae as a carbon source for mussels (e.g. [20]). Second, the animals are using increasing amounts of ^13^C depleted metabolic CO_2_. Third, factors affecting biological production may have changed. Specifically, an increase in upwelling would result in an increase in nutrient-rich, ^13^C-depleted seawater and would be consistent with our declining shell δ^13^C. Alternatively, increased respiration and decreased phytoplankton abundance could also contribute to the observed decline in shell δ^13^C.

**Figure 3:**
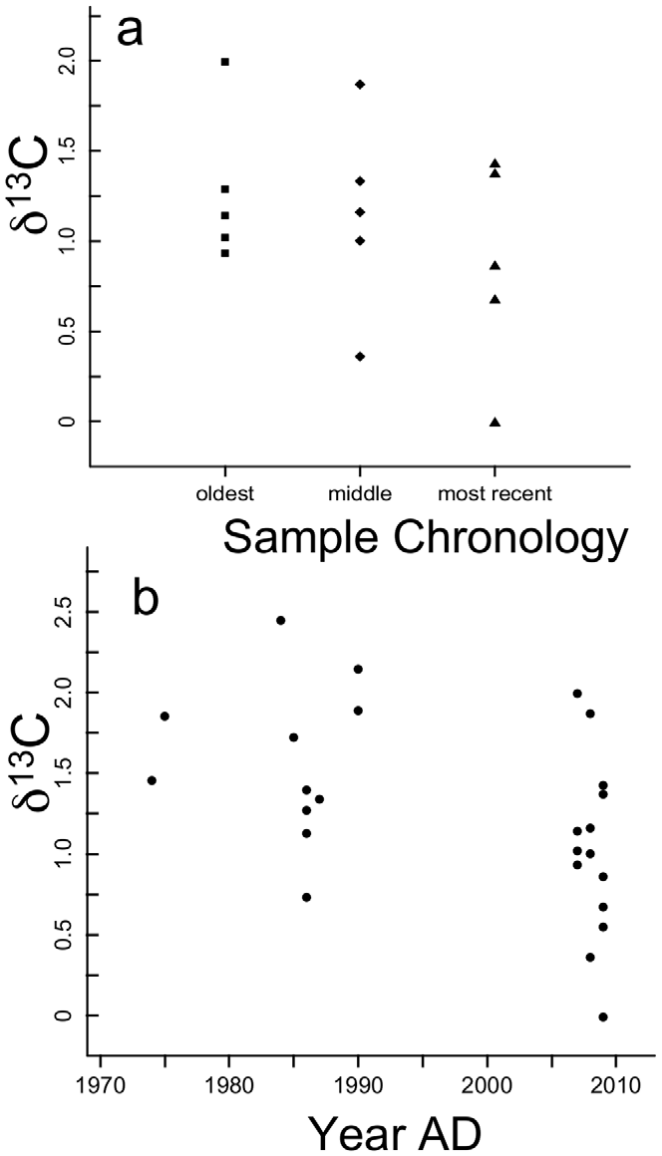
The δ^13^C (‰, PDB scale) of limpets (*Lottia pelta*) at Tatoosh Island. a. The δ^13^C of limpets collected in 2009 (n = 5) showed a significant decline among the 3 sample chronologies (−0.206 ‰ yr^−1^, p = 0.009), b. Archival and modern *L. pelta* δ^13^C values where year could be assigned with certainty, had a coefficient of −0.023 ‰ yr^−1^ (p = 0.063, n = 13) .

A change to the food web from dominance by isotopically heavier benthic algae to lighter phytoplankton could result in a pattern of decline in δ^13^C of filter-feeding species such as mussels. Because algal δ^13^C can differ by an order of magnitude more than the shell changes we saw, it would be possible to see shifts in the shell signature with relatively small dietary changes. However, such a shift would not affect benthic grazers such as the limpet *Lottia pelta*, as their diets are restricted to benthic microalgae. The significant decline in the δ^13^C of limpets over the three time points provides evidence counter to a food web shift hypothesis (Fig 3a, linear mixed effects model, time coefficient = −0.206, p = 0.009, n = 5). Although a slope coefficient of −0.206‰ yr^−1^ is greater than that of *M. californianus*, the time interval (and thus units) for that slope is uncertain because annual bands are not always distinct on *L. pelta* and the slope thus represents only a relative change among three layers. The δ^13^C of archival shells from the 1970s and 1980s (n = 7) were significantly greater than shells collected in 2009 (n = 10) (mean±se = 1.56 ‰ ±0.16 vs 1.02‰ ±0.16, F_1,15_ = 5.102, p = 0.039). When we used known years in all *L. pelta* collections by examining only the most recently produced shell layer, we estimated a slope of −0.023 ‰ yr^−1^ from ∼1974 to 2009 (p = 0.064, n = 13). The concordance in the decline of both mussel and limpet shell δ^13^C suggest that the causal process driving a decline in shell δ^13^C is unrelated to trophic mode. Further, the relatively small offset between our shell δ^13^C with that of seawater in the surface ocean (δ^13^C_DIC_  = 1.8 to 2.2 ‰ at Station P of the northeast Pacific (18)) suggests that shells reflect inorganic carbon in the water and that food web effects of highly depleted values for macroalgal versus phytoplankton [20] are unlikely to differentially affect shell values.

Although a decline in modern bivalve shell δ^13^C has been shown to be related to increased use of metabolic CO_2_ with age [21], [22], neither our midden nor archival mussel shells showed a decline related to age (Fig 1a), nor did shell of different aged individuals laid down in the same year differ in the δ^13^C (Fig 1b). Furthermore, we found no relationship between δ^13^C and δ^18^O (linear mixed effect model, coefficient  = −0.012, df = 82, p = 0.925) when we structured the analysis by individual, a result that might have indicated metabolic effects on carbon and oxygen isotopes (e.g. [23]). Thus, even though mussels may not be precipitating calcium carbonate in equilibrium with seawater [6], we cannot yet identify a metabolic process that is causing a δ^13^C decline through time in modern shells.

Changes to δ^13^C could be due to factors affecting biological production, either via changes to upwelling or related changes to productivity. Increased upwelling results in decreased seawater δ^13^C as a result of the oxidation of biological material that is isotopically light. Other studies of plant and animal tissue have attributed declines in δ^13^C that are greater than the expected based on the Seuss effect to upwelling [11], [24]; however, upwelling has not been rigorously tested. Several sources of data test the hypothesis that upwelling increased in the region, resulting in declining δ^13^C. We found a linear positive trend in the daily UI from 1998 through 2009 for 48°N, with a slope of 0.0109 (p<0.001, r^2^ = 0.016, Figure S1). When we analyzed the spring and summer period when upwelling occurs (April through September), there is no increasing trend (−0.0001, p = 0.884); the positive trend comes from an analysis of October through March only (0.021, r^2^ = 0.039, p<0.001) when downwelling occurs. Thus, it appears that it is not stronger upwelling during the season when positive values for the Upwelling Index prevail, but rather a weakening of downwelling typical of fall and winter months.

If net upwelling has increased, nitrate and soluble reactive phosphorus (henceforth “phosphate”) levels should increase as well. The concentrations of nitrate and phosphate have a known empirical relationship with δ^13^C at 42^o^N in the northeast Pacific, following Redfield ratios for both nitrate and phosphate [25]. We thus assumed a Redfield ratio and estimated whether increasing nutrients associated with upwelling can corroborate the decline in δ^13^C. Using annually averaged water nutrient data from Tatoosh, we found no statistically significant temporal trends in nitrate and phosphate (nitrate: F = 1.20, p = 0.302, slope = 0.312 µM yr^−1^; phosphate: F = 0.50, p = 0.497, slope = 0.019 µM yr^−1^, Table 1). Nonetheless, one can use these slopes to explore how nutrient changes might have affected δ^13^C. If we assume that the relationship between nitrate and phosphate through time was significantly different from zero, and used our estimated coefficients, δ^13^C is predicted to decrease 0.019 and 0.018 ‰ yr^−1^ due to increasing nitrate and phosphate, respectively, an amount that still does not explain the observed decline. A 7.13 µmol/kg mean change in nitrate over the study period (or 0.446 µmol/kg mean change in phosphate) is required to explain the decreasing δ^13^C, a change in nitrate that is not supported by our data.

We further hypothesized that any circulation pattern that might change the supply of CO_2_ in shallow waters would be revealed by temporal trends in seawater temperature surrounding Tatoosh Island. Because Tatoosh temperature is only recorded from April to September, we used Cape Elizabeth Buoy (NDBC Buoy 46041, www.ndbc.noaa.gov) for mean daily sea surface temperature (SST, °C). We found a linear negative trend in SST of −0.0002 per year from 1998 through 2009 showing temperatures declining over the past decade (Figure S2, p<0.001, r^2^ = 0.014). Although this buoy is 50 km to the southwest of Tatoosh and records SSTs several degrees higher than Tatoosh, it still serves as an indicator of relative temperature in the region [26].

Changes in the upwelling or circulation regimes could affect dissolved oxygen (DO) in these regions [4], [27]. Decreases through time in DO might indicate increased oxidation of organic matter and thus the increased release of relatively ^13^C depleted CO_2_. Although the DO probe was not functional at all times and all years at Tatoosh (Table 1), there were still 6 years of data spanning 2000 to 2009. The mean daily DO showed a positive linear trend with time (slope = 0.0008, p<0.001, r^2^ = 0.222, df  = 18304), not the negative trend that is needed to explain a declining δ^13^C associated with water influenced by high respiration.

A final possibility for the decline in δ^13^C due to biological production changes is phytoplankton decline and thus a reduced uptake of CO_2_, a hypothesis that had no support from remote sensing data that estimates chl *a* as a proxy for phytoplankton concentration (SeaWiFS (1998–2007) and MODIS data (Aqua MODIS years: 2000–2009, http://coastwatch.pfeg.noaa.gov). For SeaWiFS the monthly mean of chl *a* (mg m^−3^) of 5, 0.1 degree gridded areas around Tatoosh from 1998 to 2007 showed no linear change through time (Fig S3, slope  = −.073, p = 0.622, r^2^ = 0), a result paralleled by Aqua MODIS data on chl fluorescence (Fig S4, −0.0005, p = 0.487, r^2^ = 0). Thus, although there is some indication of global declines in phytoplankton [28], there was no indication of this pattern in the area surrounding Tatoosh.

Finally, we note that the equilibration time of the δ^13^C of seawater is about 10 times longer than that of pCO_2_, potentially decoupling the δ^13^C of DIC from surface ocean nutrient and pCO_2_ values. Sea surface δ^13^C increases with latitude [29], so the observed decrease in the shell δ^13^C may in part be attributable to increased influence of lower-latitude water over time, as previously suggested by Ortiz et al [25]. Additionally, decreasing shell δ^13^C at Tatoosh Island is consistent with upwelling of younger water, as if fossil fuel CO_2_ were invading the coastal waters both from the atmosphere and, increasingly, through the thermocline. However, the magnitude of the δ^13^C change recorded in mussel shells (−0.71 ‰ per decade) is much greater than the observed 0.15 ‰ per decade decrease for global ocean seawater [18] suggesting this cannot be a sole mechanism. Thus, despite the number of hypotheses we were able to test (Table 3), we can only speculate about sources of seawater with lower δ^13^C DIC. However, our analyses highlight the need for increased research efforts on the carbon cycle in nearshore environments (Table 3), including δ^13^C and DIC sampling, and attention to biological and circulation changes in nearshore environments.

**Table 3:** The hypothesized drivers of a declining δ^13^C in the California mussel, our hypothesis testing, and suggested further research.

Hypothesized Driver	Test of hypothesis	Suggested future research
1. A shift in the food web	A similar documented decline in limpet shell δ^13^C	Further analysis of shelled animals with differing trophic modes
2. Increased use of metabolic CO_2_ with age	Archival and midden mussels did not show a decline. Mussels of different ages record statistically indistinguishable δ^13^C values.	Increased use of metabolic CO_2_ with age remains a possibility if animals have become increasingly stressed. The analysis of shell material in mesocosm manipulations of CO_2_ and food availability would reveal the relationship between increasing pCO_2_ of seawater and physiological changes that are incorporated into the shell.
3. Changes to biological production	There are no significant changes to upwelling, dissolved oxygen, nutrients, sea surface temperature, or chlorophyll.	Increased nearshore monitoring patterns for pCO_2_, seawater δ^13^C , phytoplankton abundance, and nutrients. Many current monitoring and remote sensing programs are far from coastal habitats.

### Conclusions

In sum, we found no evidence that a single factor accounts for the rapid decline that we observed in δ^13^C of *M. californianus* shells. Although atmospheric CO_2_ explains a slope of −0.028 ‰ yr^−1^, the remaining changes seems unrelated to the type of carbon available to filter feeders. Environmental variables that might have contributed to declining shell δ^13^C over the past decade included an increase in upwelling and a possible increase in nitrate and phosphate. However, these changes are slight and unable to account for a slope 0.043‰ per year lower than expected based on calculations using atmospheric CO_2_ concentrations. Upwelling, for example, is increasing only 0.0109 units per year over the past decade - a change that seems inconsequential given that the UI can range over hundreds of units over several days. Similarly, the nitrate and phosphate increases that would have to be inferred over this interval to have so greatly affected δ^13^C (7.13 and 0.446 µmol/kg, respectively) have not been observed in this area.

Our observed decline in shell δ^13^C parallels other signs of rapid changes to the nearshore carbon cycle in the northeast Pacific, including a decline in pH that is an order of magnitude greater than predicted by an equilibrium response to the rising CO_2_ concentration of the atmosphere [2], the presence of low pH water throughout the region [3], [4], and a record of a similarly steep decline in δ^13^C of algal calcium carbonate in the Gulf of Alaska [11]. Whether the decline we report can be attributed to single factors such as circulation changes that have not yet been documented, a fundamental difference in the cycling of carbon in this region, an alteration of carbon metabolism in these animals, perhaps via an effect of changing seawater carbon chemistry on shell composition (e.g. [8]), or undescribed interactions among these factors is unknown. Mussel shells, however, indicate that these changes are unprecedented and the lack of a clear causal variable underscores the need for better quantifying carbon dynamics in nearshore environments.

## Materials and Methods

In April of 2009 24 of the largest *M. californianus* were collected in the mid intertidal (tidal height ∼0.6–1.0 m above MLLW) in the area of Tatoosh Island, WA known as Hedophyllum Cove, where they recruited in 1999 and 2000 [30], approximately coincident with our instrument deployment in the same locale. An additional 9 small mussels (38.8–52.6 mm) were also collected to test whether younger mussels had similar isotopic values for a given year. We also collected 3 of the largest *M. californianus* we could find on Tatoosh in 2009, at a site known as the Glacier on Tatoosh and 8 of the largest shells from the Finger site and 3 from the Main Beach of Tatoosh. In April 2010, we collected 7 more of the largest available animals from Hedophyllum Cove to sample only in the 2009 growth area. To test whether mussels from a site 85 km east in the Strait of Juan de Fuca showed similar isotopic ratios of carbon and oxygen to Tatoosh, 10 of the largest shells from the mid intertidal of Observatory Point (48.264°N, 124.248°W) were collected coincident with the April 2009 collection on Tatoosh.

To prepare the shells for isotope sampling, we removed all soft parts and epiphytes from the shells and dried them at 40°C. Our methods were based on Schöne et al. [31] where shells were cleaned in a 5% bleach solution for 1h, then rinsed three times in MilliQ water for 15 min. The length, height and width of each shell was measured, then we sectioned one valve of each individual lengthwise along its axis of maximum growth by first reinforcing with epoxy (JB QuikWeld, Sulphur Springs, TX), then sawing with a Isomet™ Low Speed Saw and diamond edge cutting blades (Buehler Co.). We prepared a cross sectional slide (1.5 mm width) along the axis of maximum growth. We polished the slides using a 600 grit silicon carbide powder (Buehler Co.) followed by a microcloth with 3 µm diamond polishing suspension (MetaDi Polycrystalline Diamond Suspension, Buehler Co.) to better visualize the annual bands. *M. californianus* deposits calcitic shell in annual layers with the newest shell material deposited in proximity to the inner soft tissue of the animal. Our analyses focused on the calcitic inner prismatic layers [32]; mussels were aged by counting the number of these bands designating the band closest to the mantle as the most recent. The fact that the Hedophyllum Cove shells showed ∼10 annual bands and were known to have recruited in 1999 [30] gave us confidence in our aging method. Shell material was collected by drilling in the center of each growth band in each individual using a Dental Rotary drill (Brasseler USA). Because we knew the date of shell collection, we were able to assign growth increments to particular years. Shell material was analyzed for stable isotopes of carbon (δ^13^C) and oxygen (δ^18^O) in the Earth Systems History Laboratory at Brown University (Providence, RI).

Sources of archival shells included 9 shells collected in 1986, 1987, 1990 from Oystercatcher (*Haematopus bachmani*) middens (JTW), 8 shells collected by E. Sanford in August 2001 at north-facing part of Tatoosh, and 5 shells collected by T. Suchanek in the 1970s. The precise date of collection of the shells is not known, though we placed the collection date at 1975 in the midst of the time that Suchanek was studying mussels on Tatoosh. Tatoosh was a Native American summer camp for the Makah for centuries prior to occupation by the US Coast Guard and Navy in the 1800s. Middens have been excavated on the island [14] and we obtained 11 *M. californianus* valves from the Makah Cultural and Resource Center. The midden shells were prepared with identical methodology except that the entire valve was encased in epoxy prior to sawing to protect these older shells from breakage. Shell ages were obtained from NOSAMS (www.nosams.whoi.edu) via radiocarbon dating by sampling each shell individually in the last complete annual growth band recorded by the animal.

The limpet, *Lottia pelta*, is an abundant benthic grazer in the rocky intertidal of the northeast Pacific. Limpets add new calcium carbonate throughout the interior of the shell, and grow from the apex outward; hence, the apex provides the longest time series of calcium carbonate deposition. Limpets (*Lottia pelta*) were collected at several sites on Tatoosh in 2009; archival specimens were from oystercatcher middens described above (1986, 1987, 1990), while a single individual came from the 1970s collection of Suchanek. We sectioned limpets through the apex using the methods described above. Because limpets erode at the apex, limpets did not provide sufficiently high resolution for annual sampling. Instead, the apex was sampled in presumed time horizons of the most recent year of growth, and back through time for a midpoint and an ‘oldest’ horizon. We thus only used limpets for a relative trend in isotopic signature and only assigned the most recent growth strata to a particular year.

Instrumental data on seawater temperature, salinity, pH, dissolved oxygen, and chl *a* have been recorded every 30 min during the spring and summer months since 2000 at Tatoosh Island. Detailed methods on this collection have been described [2], and have continued through the period of growth recorded by the *M. californianus* shells. We have also measured water nutrients (nitrate, nitrite, ammonium, soluble reactive phosphorus, silicate) monthly at 10 locales around Tatoosh Island from April to September since 1998.

Environmental variables estimated over larger spatial and temporal scales included the upwelling index (UI) for 48°N, 125°W (http://www.pfeg.noaa.gov), an indicator of upwelling strength based on wind stress measurements, as well as the Pacific Decadal Oscillation (PDO, http://jisao.washington.edu/pdo/PDO.latest), a composite indicator of ocean temperature anomalies [33], seawater temperature from Buoy 46041 ∼50 km to the southwest from Tatoosh (www.ndbc.noaa.gov), and remote sensing of chl *a* (SeaWiFS, AquaModis).

Isotopes of carbon and oxygen were sampled on an annual time scale while all environmental data was finer-grained, ranging from half-hourly to monthly. We thus aggregated environmental data, estimating annual means for our instrumental data that spanned April to September. For the UI and PDO we averaged both over the entire year or using only the April to September period corresponding to our measurements. These data groupings represented two different growth models for these molluscs – one where they grew year-round, and another where the principal growing season for *M. californianus* is the spring and summer (e.g. [34]). In testing for relationships among environmental factors and δ^13^C and δ^18^O, the plethora of variables would have compromised our degrees of freedom. We thus structured our analyses to focus on those factors that are known to have a relationship to each element, tested their relationship in isolation, and then used a multi-effect model where warranted. Our statistical analyses recognized the structure of data, where individuals are sampled multiple times and thus may impart a correlation structure on the data. We thus treated shell identity as a random effect in mixed effect models when analyzing time trends and relationships with environmental variables. All statistics were done with R (www.r-project.org/).

We obtained the necessary permits for acquiring shells including a permit from the Makah Tribal Nation (for Tatoosh collections) and Washington Department of Fish and Wildlife shellfishing permit for collections at Observatory Point.

## Supporting Information

Figure S1 **Daily Upwelling Index (UI) in proximity to Tatoosh Island (48°N) from 1998–2009.** The UI showed a significant positive linear trend (slope = 0.0109, p<0.001), though the r^2^ was low (r^2^ = 0.016).
(TIF)

Figure S2 **Mean daily SST for Buoy 46041 (Cape Elizabeth) in °C from 1998–2009.** The SST showed a significant negative trend (slope = −0.0002, p<0.001), though the r^2^ was low (r^2^ = 0.014).
(TIF)

Figure S3 **Mean monthly SeaWiFS values from 1998–2007 (chl** ***a*** **in mg m^−3^).** There was no significant pattern for SeaWiFS with time (−0.073, p = 0.622).
(TIF)

Figure S4 **Mean monthly Aqua MODIS chl fluorescence data from 2000–2009.** There was no significant pattern for AquaMODIS data with time (−0.0005, p = 0.487).
(TIF)
