# Supplementary figures and images for: Rapid Environmental Change over the Past Decade Revealed by Isotopic Analysis of the California Mussel in the Northeast Pacific
Source: PLoS One. 2011 Oct 3;6(10):e25766. doi: 10.1371/journal.pone.0025766 (PMC3185010; doi:10.1371/journal.pone.0025766)

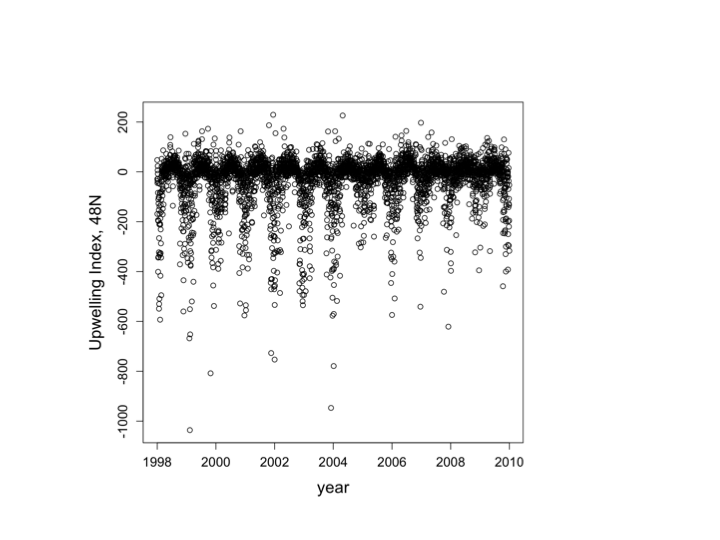

Supplement: Figure S1 — Daily Upwelling Index (UI) in proximity to Tatoosh Island (48°N) from 1998–2009. The UI showed a significant positive linear trend (slope = 0.0109, p<0.001), though the r2 was low (r2 = 0.016). (TIF) [file pone.0025766.s001.tif]

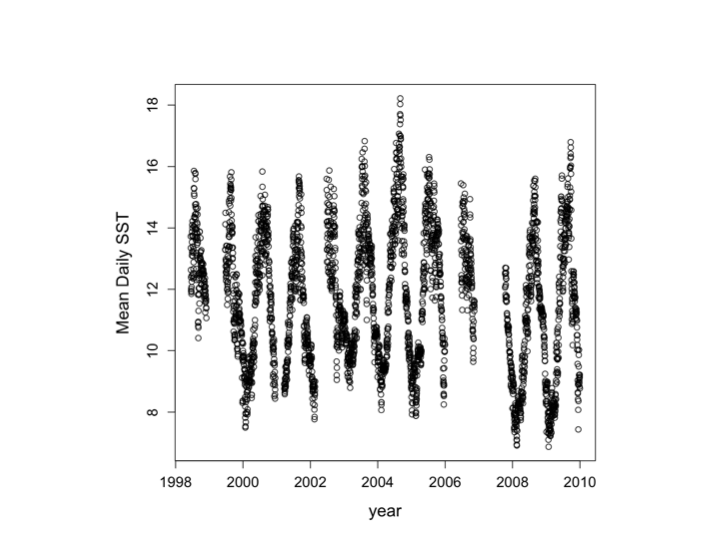

Supplement: Figure S2 — Mean daily SST for Buoy 46041 (Cape Elizabeth) in °C from 1998–2009. The SST showed a significant negative trend (slope = −0.0002, p<0.001), though the r2 was low (r2 = 0.014). (TIF) [file pone.0025766.s002.tif]

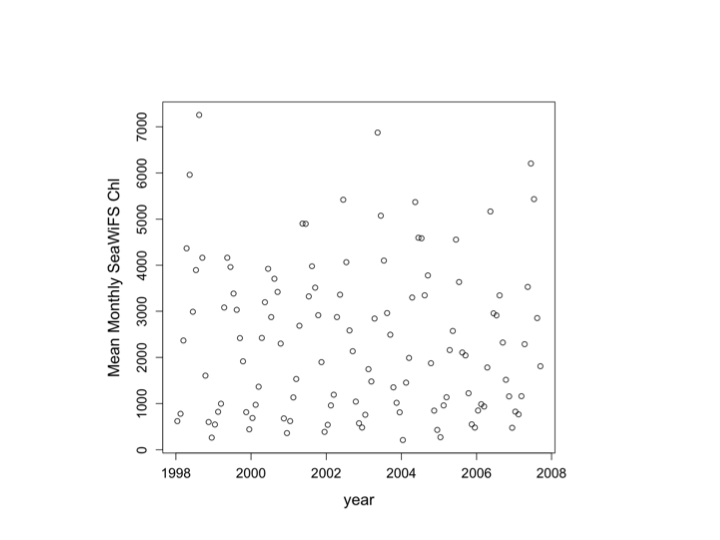

Supplement: Figure S3 — Mean monthly SeaWiFS values from 1998–2007 (chl a in mg m−3). There was no significant pattern for SeaWiFS with time (−0.073, p = 0.622). (TIF) [file pone.0025766.s003.tif]

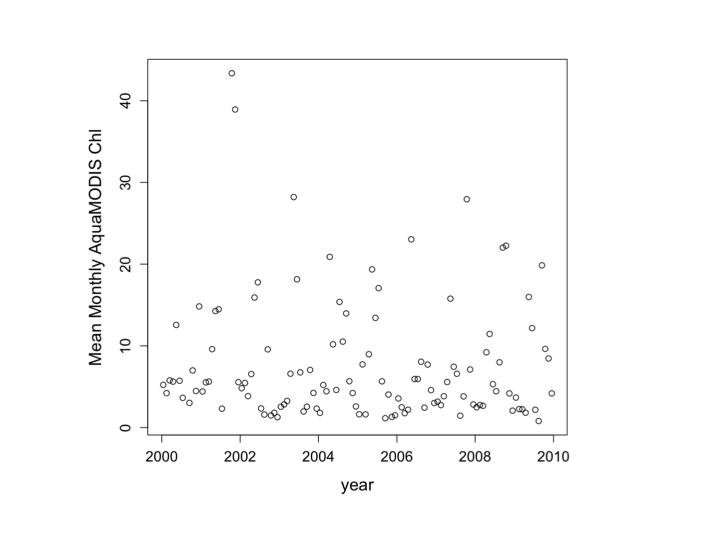

Supplement: Figure S4 — Mean monthly Aqua MODIS chl fluorescence data from 2000–2009. There was no significant pattern for AquaMODIS data with time (−0.0005, p = 0.487). (TIF) [file pone.0025766.s004.tif]
